# Adenosine A_2A_ Receptor in Bone Marrow-Derived Cells Mediated Macrophages M2 Polarization *via* PPARγ-P65 Pathway in Chronic Hypoperfusion Situation

**DOI:** 10.3389/fnagi.2021.792733

**Published:** 2022-01-03

**Authors:** Ke-Jie Mou, Kai-Feng Shen, Yan-Ling Li, Zhi-Feng Wu, Wei Duan

**Affiliations:** ^1^Department of Neurosurgery, Bishan Hospital of Chongqing, Chongqing, China; ^2^Department of Neurosurgery, Xinqiao Hospital, Army Medical University, Chongqing, China; ^3^Department of Neurology, Xinqiao Hospital, Army Medical University, Chongqing, China; ^4^Department of Pediatrics, Xinqiao Hospital, Army Medical University, Chongqing, China

**Keywords:** adenosine A_2A_ receptor, microglia/macrophages polarization, PPARγ, P65, inflammatory factors

## Abstract

**Background:** The role of adenosine A_2A_ receptor (A_2A_R) in the ischemic white matter damage induced by chronic cerebral hypoperfusion remains obscure. Here we investigated the role of A_2A_R in the process of macrophage polarizations in the white matter damage induced by chronic cerebral hypoperfusion and explored the involved signaling pathways.

**Methods:** We combined mouse model and macrophage cell line for our study. White matter lesions were induced in A_2A_R knockout mice, wild-type mice, and chimeric mice generated by bone marrow cells transplantation through bilateral common carotid artery stenosis. Microglial/macrophage polarization in the corpus callosum was detected by immunofluorescence. For the cell line experiments, RAW264.7 macrophages were treated with the A_2A_R agonist CHS21680 or A_2A_R antagonist SCH58261 for 30 min and cultured under low-glucose and hypoxic conditions. Macrophage polarization was examined by immunofluorescence. The expression of peroxisome proliferator activated receptor gamma (PPARγ) and transcription factor P65 was examined by western blotting and real-time polymerase chain reaction (RT-PCR). Inflammatory cytokine factors were assessed by enzyme-linked immunosorbent assay (ELISA) and RT-PCR.

**Results:** Both global A_2A_R knockout and inactivation of A_2A_R in bone marrow-derived cells enhanced M1 marker expression in chronic ischemic white matter lesions. Under low-glucose and hypoxic conditions, CGS21680 treatment promoted macrophage M2 polarization, increased the expression of PPARγ, P65, and interleukin-10 (IL-10) and suppressed the expression of tumor necrosis factor-α (TNF-α) and interleukin-1β (IL-1β). The CGS21680-induced upregulation of P65 and IL-10 was abolished in macrophages upon PPARγ knockdown. The downregulation of TNF-α and IL-1β by CGS21680 was less affected by PPARγ knockdown.

**Conclusions:** In the cerebral hypoperfusion induced white matter damage, A_2A_R signaling in bone marrow-derived cells induces macrophage M2 polarization and increases the expression of the anti-inflammatory factor IL-10 *via* the PPARγ-P65 pathway, both of which might explain its neuroprotective effect.

## Introduction

The role of adenosine A_2A_ receptor (A_2A_R) in ischemic brain injury has attracted increasing attention in recent years. It has been shown that A_2A_R knockout (KO) could significantly inhibit the local inflammatory response and ameliorate acute ischemic brain injury (Chen et al., 1999). However, we found that A_2A_R KO significantly enhanced the local inflammatory response and aggravated ischemic white matter injury induced by chronic cerebral hypoperfusion (Duan et al., 2009). Therefore, the pathological mechanisms of the ischemic white matter lesion induced by chronic cerebral hypoperfusion remain obscure and the detailed effects of A_2A_R on ischemic brain injury are still unclear. Interestingly, a recent research found that selective inactivation of A_2A_R in bone marrow-derived cells (BMDCs) promotes inflammatory cytokine expression and aggravates chronic hypoperfusion-induced white matter lesions (Ran et al., 2015), suggesting that A_2A_R in BMDCs might play a crucial role in such type of white matter injury. In line with this result, activation of A_2A_R was found to inhibit inflammatory injury in peripheral organs (e.g., the lung and liver), and peripheral macrophages might be the key inflammatory cells involved in the aggravation of white matter injury induced by chronic cerebral hypoperfusion (Patel et al., 2020; Wang et al., 2020). Therefore, further studies are needed to clarify the role of A_2A_R in BMDCs in regulating inflammatory responses and white matter injury induced by chronic hypoperfusion.

The release of inflammatory factors is determined by the state of inflammatory cells (Ahmad et al., 2017a, 2018b, 2019; Ansari et al., 2017a; Lan et al., 2017). Resident microglia and peripheral macrophages are rapidly mobilized to the site of injury and initiate the release of effective molecules and recruitment of other immune cells. Microglia and macrophages are highly plastic cells that can adopt diverse phenotypes and activate different functional programs in response to specific microenvironmental signals, thus been implicated in the pathology of numerous diseases involving acute ischemic brain injury (Hu et al., 2012; Qin et al., 2017), multiple sclerosis (Chu et al., 2019), spinal cord injury (Paterniti et al., 2011), Alzheimer's disease, and other central nervous system associated diseases (Hu, 2020; Lei et al., 2021). However, the modulating role of A_2A_R in microglia/macrophage polarization in the development of white matter injury has not yet been comprehensively characterized.

In this study, we investigated the effect of A_2A_R on microglia/macrophage polarization with a mouse model of chronic cerebral hypoperfusion induced white matter lesions by bilateral common carotid artery stenosis (BCAS). Furthermore, we established chimeric mice by BMDCs transplantation to analyze the role of A_2A_R in bone marrow derived microglia/macrophage polarization after cerebral hypoperfusion. In addition, *in vitro* cell line study was performed to verify the effect of A_2A_R on macrophage polarization and explore related molecular mechanisms.

## Materials and Methods

### Animals

The A_2A_R KO C57BL/6 mice were a gift from Dr. Jiang-Fan Chen (Boston University School of Medicine, Boston, MA). The genotype of each mouse was determined using polymerase chain reaction (PCR), as previously reported (Chen et al., 1999). Age-matched KO and wild-type (WT) mice (10 weeks old) were used for this study. The mice were housed under standard conditions (temperature: 23 ± 1°C; illumination: 12-h light/12-h dark cycle; food and water: *ad libitum*). All surgeries were performed under anesthesia with sodium pentobarbital (50 mg/kg). All animal care and experimental procedures were approved by the Institutional Animal Care and Use Committee of the Army Medical University (SYXK-PLA-2007035), where the principle of the 3Rs (**R**eplacement, **R**eduction, and **R**efinement) has been developed and enhanced.

### Generation of Chimeric Mice

Chimeric mice were generated *via* BMDC transplantation as previously reported (Yu et al., 2004; Ran et al., 2015). Male recipient mice were irradiated with a total dose of 12.5 Gy of ^60^Co. Bone marrow cells were isolated from female donor mice after scarification with a lethal dose of sodium pentobarbital. Recipient male mice were injected with an aliquot of ~2 × 10^8^ BMDCs in 300 μl RPMI-1640 medium containing 10% fetal bovine serum *via* the tail vein. The efficiency of selective reconstitution of BMDCs in the chimeric mice was assessed seven weeks after the transplantation according to previous studies (Yu et al., 2004; Ran et al., 2015).

### Establishment of Chronic Cerebral Hypoperfusion by BCAS

BCAS was performed according to the previously established methods (Shibata et al., 2004). The microcoils (Invitrotech, Osaka, Japan) used for BCAS were composed of piano wire with an inner diameter of 0.18 mm. The bilateral common carotid arteries of the mice were exposed through a midline cervical incision and freed from their sheaths after anesthetization. Subsequently, the right artery was gently placed between the loops of the microcoil directly beneath the carotid bifurcation and was wrapped around the common carotid artery. After 30 min, another microcoil of the same size was wrapped around the left common carotid artery. The rectal temperature was maintained between 36.5 and 37.5°C throughout the procedure. Cerebral blood flow was monitored by laser Doppler flowmetry as previously described (Shibata et al., 2004). In sham group, the common carotid arteries were exposed but not wrapped by microcoil.

### Immunofluorescence Staining

Mice were deeply anesthetized with pentobarbital and transcardially perfused with PBS followed by 4% paraformaldehyde at 3, 7, 14, and 28 d after BCAS. The brains were harvested, postfixed in 4% paraformaldehyde for 12 h, and subsequently stored in 30% sucrose. Serial coronal sections (30 μm) spanning the anterior region of the callosum (bregma-0.26 mm) to the anterior region of the hippocampus (bregma-0.94 mm) were cut with a cryostat. The serial coronal sections were preincubated in 5% goat serum and incubated overnight at 4°C in the primary antibody solution followed by three-time washes in the PBS. The sections were then incubated in the secondary antibody solution for 1 h at room temperature and PBS-washed. Finally, the sections were evaluated by confocal microscopy. The following primary antibodies were used in the study: anti-inducible nitric oxide synthase (iNOs) antibody (M1 polarization marker, 1:200, NB300-605, NOVUS Biologicals, Building IV Centennail, CO 80112,USA), goat anti-CD16/32 antibody (M1 polarization marker, 1:200, AF1460, R&D Systems, Minneapolis, Toll Free USA, Canada), rabbit anti-Arginase-1 (Arg-1) antibody (M2 polarization marker, 1:200, NBP1-32731, NOVUS Biologicals, Building IV Centennial, CO 80112, USA), rabbit anti-CD206 antibody (M2 polarization marker, 1:100, NBP1-90020, NOVUS Biologicals, Building IV Centennial, CO 80112, USA) and rabbit anti-Iba1 antibody (1:200, ab178847, Abcam, Waltham, MA 02453, USA), and the following secondary antibodies were used: fluorescent-conjugated sheep anti-rabbit or anti-goat IgG (1:50, ZF0311; ZF0314; ZF0316; ZF0317, Zhongshan JinQiao Biotechnology Co., Ltd, Beijing, China).

For confocal microscopy detection, 200× non-overlapping high-power fields (0.5 × 0.5 mm, total area of 0.25 mm^2^) in the center of the corpus callosum were selected using a square grid inserted into the eyepiece. The integrated optical density (IOD) of the target protein in the corpus callosum in five mice from each group was analyzed using the Image-Pro Plus 4.5 (Media Cybernetics, Silver Spring, MD). The IODs in the regions of interest were measured as gray values. Three fields within the regions of interest in three sections per animal were examined. For each section, the IODs in three selected fields of the corpus callosum were averaged.

### Cell Culture and Stimulation

The RAW264.7 macrophage cell line was purchased from ScienCell Laboratory (San Diego, California, USA) and cultured in the DMEM with 10% FBS (Gibco, USA), 100 U/mL penicillin, and 100 μg/mL streptomycin at 37°C in 5% CO_2_ in a humidified incubator. For the A_2A_R manipulation experiment, 1 × 10^6^ macrophage cells per well were treated by the A_2A_R agonist CGS21680 (1.0 μM) or antagonist SCH58261 (1.0 μM) for 30 min and transferred to the low-glucose DMEM and cultured under a hypoxic condition (1% O_2_, 5% CO_2_, and 94% N_2_; 37°C). For the shRNA experiment, macrophage cells were transfected with a lentivirus carrying the shRNA targeting PPARγ (5'-3': GCTGGCCTCCCTGATGAATAA). Following experiments were performed after the cells were cultured in low-glucose and hypoxic conditions for 2, 6, 12, or 24 h.

### Western Blot

Total protein was extracted from cells using a whole protein extraction kit (Key GEN, China). Total protein concentrations were determined on a UV spectrophotometer using a modified Bradford assay (Beckman Coulter, Fullerton, CA; Ran et al., 2015). Equal amounts of protein from each sample were separated *via* electrophoresis on 8% polyacrylamide gels and then transferred to polyvinylidene fluoride (PVDF) membranes. The membranes were blocked by 5% skimmed milk and incubated overnight at 4°C with the following primary antibodies: a rabbit anti-PPARγ antibody (1:1000, AF6284, Affinity Bioscience, Beijing, China), rabbit anti-P65 antibody (1:1000, NB100-2176, NOVUS Biologicals, Building IV Centennial, CO 80112, USA), or rabbit anti-p-P65 antibody (1:1000, NB100-82088, NOVUS Biologicals, Building IV Centennial, CO 80112, USA). After washed with TBST for three times, the membranes were incubated with goat anti-rabbit secondary antibody (1:1000, ZB2301, ZhongShan JinQiao Biotechnology Co., Ltd, Beijing, China) or goat anti-mouse secondary antibody (1:100, ZB2305, ZhongShan JinQiao Biotechnology Co., Ltd, Beijing, China). The amount of β-actin (detected by anti-beta-actin, 1:2000, SC-47778, Santa Cruz Biotechnology, Santa Cruz, CA) was used as the internal control. The PVDF membranes were developed and visualized, and the optical density (OD) of each specific protein band was measured using an image analysis software (QuantityOne 4.4.0.36; Bio-Rad, Hercules, CA) and normalized to the OD of the β-actin.

### Real-Time Quantitative PCR (RT-PCR)

Total RNA was isolated from macrophages and reversely transcribed using MMLV Reverse Transcriptase (Thermo Fisher Scientific, USA). Complementary DNA was amplified by PCR using a SYBR Green kit (TaKaRa BioInc, Dalian, China) and an ABI 7500 qPCR system (Applied Biosystems). Each PCR cycle consisted of 6 min of denaturation at 95°C, 30 s of denaturation at 95°C, 45 s of annealing at 65°C and 30 s of extension at 72°C, and 40 PCR cycles were performed. β-actin was used as the internal control. The 2^−ΔΔct^ method was used for the quantification. The sequences of the primers used for PPARγ, P65, inflammatory cytokines, and β-actin were shown in the Supplementary Table 1. For each sample, at least three independent PCR experiments were performed.

### Enzyme-Linked Immunosorbent Assay (ELISA)

The protein expression of inflammatory cytokines from macrophages cultured under low-glucose and hypoxic conditions was examined by ELISA. Mouse Quantikine ELISA kits for tumor necrosis factor-α (TNF-α), interleukin-1β (IL-1β), and IL-10 (R&D Systems, MTA00B, MLB00C, and M1000B) were used in the study. Working reagents were prepared and 50 μl of standard controls and cell supernatants from each group were added to the wells of a plate according to manufactures instructions. The antibody was added and incubated for two hours at room temperature, followed by incubation with the substrate solution for 30 min. The absorbance was read using a FLvostar Omega microplate reader at 450 nm and 540 nm.

### Immunocytochemistry

Briefly, cultured macrophages were fixed in 4% formaldehyde for 1 h and incubated with a rabbit anti-iNOs antibody (M1 polarization marker, 1:200, NB300-605, NOVUS Biologicals, Building IV Centennial, CO 80112, USA) or rabbit anti-Arg-1 antibody (M2 polarization marker, 1:200, NBP1-32731, NOVUS Biologicals, Building IV Centennial, CO 80112, USA) overnight at 4°C. The cells were then PBS-washed and incubated with fluorescein isothiocyanate FITC/TRITC-conjugated goat anti-rabbit secondary antibodies (1:50, ZF0311; ZF0316, Zhongshan JinQiao Biotechnology Co., Ltd, Beijing, China). After washed with PBS, the cells were stained with DAPI (40 mg/ml) for 5 min and examined with a laser-scanning microscope (ZEISS, Germany). The IODs of the target proteins were analyzed using the Image-Pro Plus 4.5 software (Media Cybernetics, Silver Spring, MD). Briefly, a Leica DMIRB microscope was used to examine each cell section, and 200× non-overlapping high-power fields (0.5 × 0.5 mm, total area of 0.25 mm^2^) in the center of the eyepiece were selected. The IODs in the regions of interest were measured as gray values, and the number of positive cells in these regions was counted. Three fields within the regions of interest in three slices from each group were examined. For each slice, the ratios of the IOD to the number of positive cells in three selected fields were averaged.

### Statistical Analysis

The data were expressed as the mean ± SEM. Two-way ANOVA test followed by Turkey's multiple comparisons test was used to detect the difference among more than two groups. All of data used for the analysis were viewed in a blinded manner. The data were plotted and analyzed with GraphPad Prism 6. *P* < 0.05 was considered statistically significant.

## Results

### A_2A_R Deletion Affected the Expression of M1 and M2 Markers in Mice With BCAS

**Figure 1:**
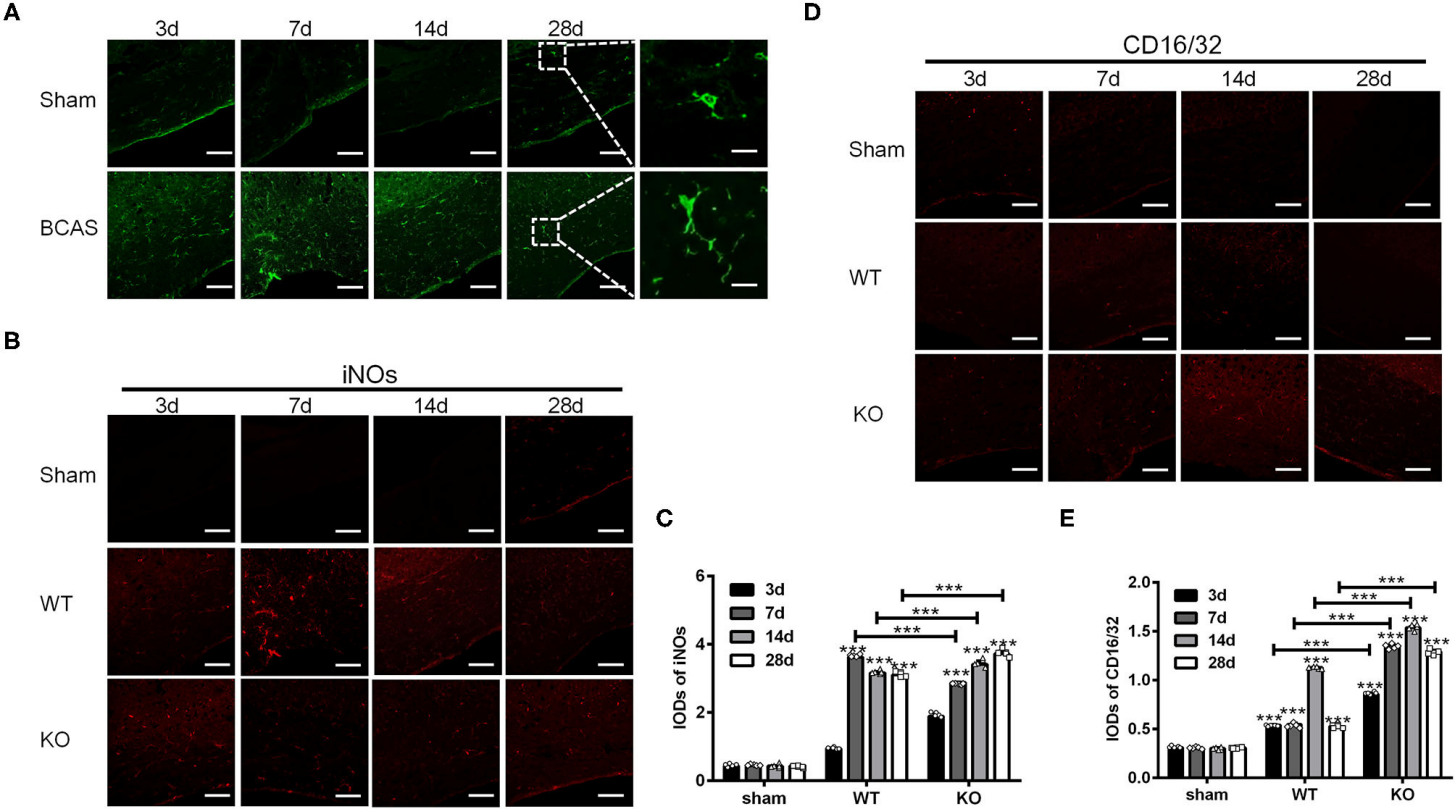
Knockout of A_2A_R enhanced the expression of iNOs and CD16/32 in corpus callosum of mice with chronic cerebral hypoperfusion. **(A)** Representative images showing the morphology of Iba1 positive glia in the corpus callosum of mice at 3, 7, 14, and 28 d after sham or BCAS operation. Scale bars: 50 and 5μm. **(B)** Representative images showing the staining of iNOs in the corpus callosum of sham operated WT mice (upper panel) and BCAS operated WT and A_2A_R KO mice at 3, 7, 14, and 28 d. Scale bars: 50 μm. **(C)** Statistically analysis showing the IODs for iNOs was increased after BCAS both in the WT and KO group Moreover, the IODs of iNOs were peaked at 7 d in the WT group and decreased at 14 and 28 d, while the IODs of iNOs in KO group were continuously increased from 3–28 d and reached a greater level than that in the WT group from 14 d (***P < 0.001, Two-way ANOVA test followed by Turkey's multiple comparisons test. *n* = 5 mice for each group). **(D)** Representative images showing the staining of CD16/32 in the corpus callosum of sham, BCAS operated WT and A_2A_R KO mice at 3, 7, 14, and 28 d. Scale bars: 50 μm. **(E)** Statistically analysis showing the IODs for CD16/32 was increased after BCAS both in the WT and KO group, and the increase in KO group were more dramatic compared to the alteration in the WT mice (***P < 0.001, Two-way ANOVA test followed by Turkey's multiple comparisons test. *n* = 5 mice for each group).

In the present study, we induced chronic cerebral hypoperfusion in A_2A_R KO mice and WT mice by BCAS and then measured the M1 and M2 markers by immunofluorescence staining in a time series manner (i.e., 3, 7, 14, and 28 d after BCAS). Glia in the corpus callosum was activated after BCAS, as indicated by the increase of Iba1 positive cells (Figure 1A). Representative immunofluorescence staining images for M1 makers including inducible nitric oxide synthase (iNOs) and CD16/32 in the corpus callosum in the sham group (WT without BCAS), WT group and KO group after BCAS were shown (Figures 1B,D). Statistical analysis showed that the IODs of iNOs were increased after BCAS both in the WT and KO groups, while the ones in the sham group remained constant (^***^P < 0.001 versus the same time point in sham group, Two-way ANOVA test followed by Turkey's multiple comparisons test; Figure 1C). Moreover, the IODs of iNOs were peaked at 7 d in the WT group and decreased later at 14 and 28 d, while the IODs of iNOs in KO group were continuously increased from 3 to 28 d and reached a greater level than that in the WT group from 14 d (^***^P < 0.001, Two-way ANOVA test followed by Turkey's multiple comparisons test; Figure 1C). Consistent with iNOs, the IODs of CD16/32 were increased both in the WT and KO groups after BCAS (Figure 1E) (^***^P < 0.001 versus the same time point in sham group, Two-way ANOVA test followed by Turkey's multiple comparisons test). However, the IODs of CD16/32 were increased more dramatically in the KO mice after BCAS compared to the alteration in the WT mice (^***^P < 0.001, Two-way ANOVA test followed by Turkey's multiple comparisons test; Figure 1D). Together, these findings suggested that the corpus callosum expression of iNOs and CD16/32 in the A_2A_R KO mice was enhanced after BCAS.

**Figure 2:**
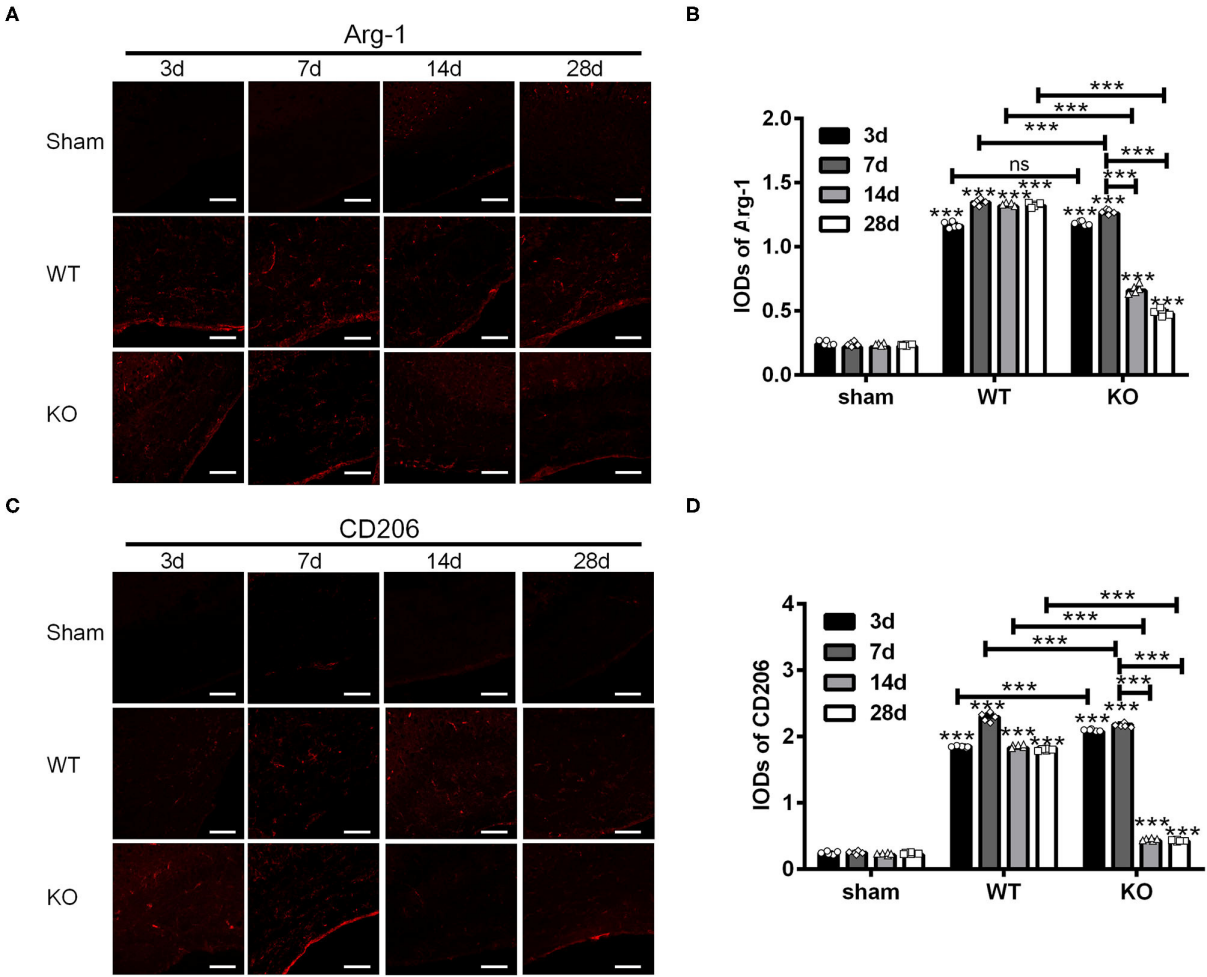
Knockout of A_2A_R reduced the expression of Arg-1 and CD206 in corpus callosum of mice underwent chronic cerebral hypoperfusion. **(A,C)** Representative images showing the expression of Arg-1 in sham, WT and KO group at 3, 7, 14, and 28 d after BCAS. Scale bars: 50 μm. **(B,D)** Statistically analysis showing the IOD for Arg-1 and CD206 was increased both in WT and KO groups after BCAS. In addition, the IODs of Arg-1 and CD206 were decreased significantly in the KO mice after reaching peak at 7 d after BCAS, while remained relatively constant in the WT mice at the same time (***P < 0.001, Two-way ANOVA test followed by Turkey's multiple comparisons test. *n* = 5 mice for each group). ns, non significant; *p* >0.05.

Next, we examined the expression of Arg-1 and CD206 in the sham, WT and KO groups in the similar way (Figures 2A,C). Statistical analysis showed that the IODs of Arg-1 and CD206 in the corpus callosum were not affected by the sham operation but increased both in the WT and KO groups after BCAS (^***^P < 0.001 versus the same time point in sham group, Two-way ANOVA test followed by Turkey's multiple comparisons test; Figures 2B,D). In addition, both the IODs of Arg-1 and CD206 were decreased significantly in the KO mice after reaching peak at 7 d after BCAS, while remained relatively constant in the WT mice at that time (^***^P < 0.001, Two-way ANOVA test followed by Turkey's multiple comparisons test; Figures 2B,D). These results suggested that the corpus callosum expression of the M2 markers Arg-1 and CD206 was impaired in the A_2A_R KO mice after BCAS.

### A_2A_R in BMDCs Regulated M1/M2 Polarization in Mice With White Matter Damage

**Figure 3:**
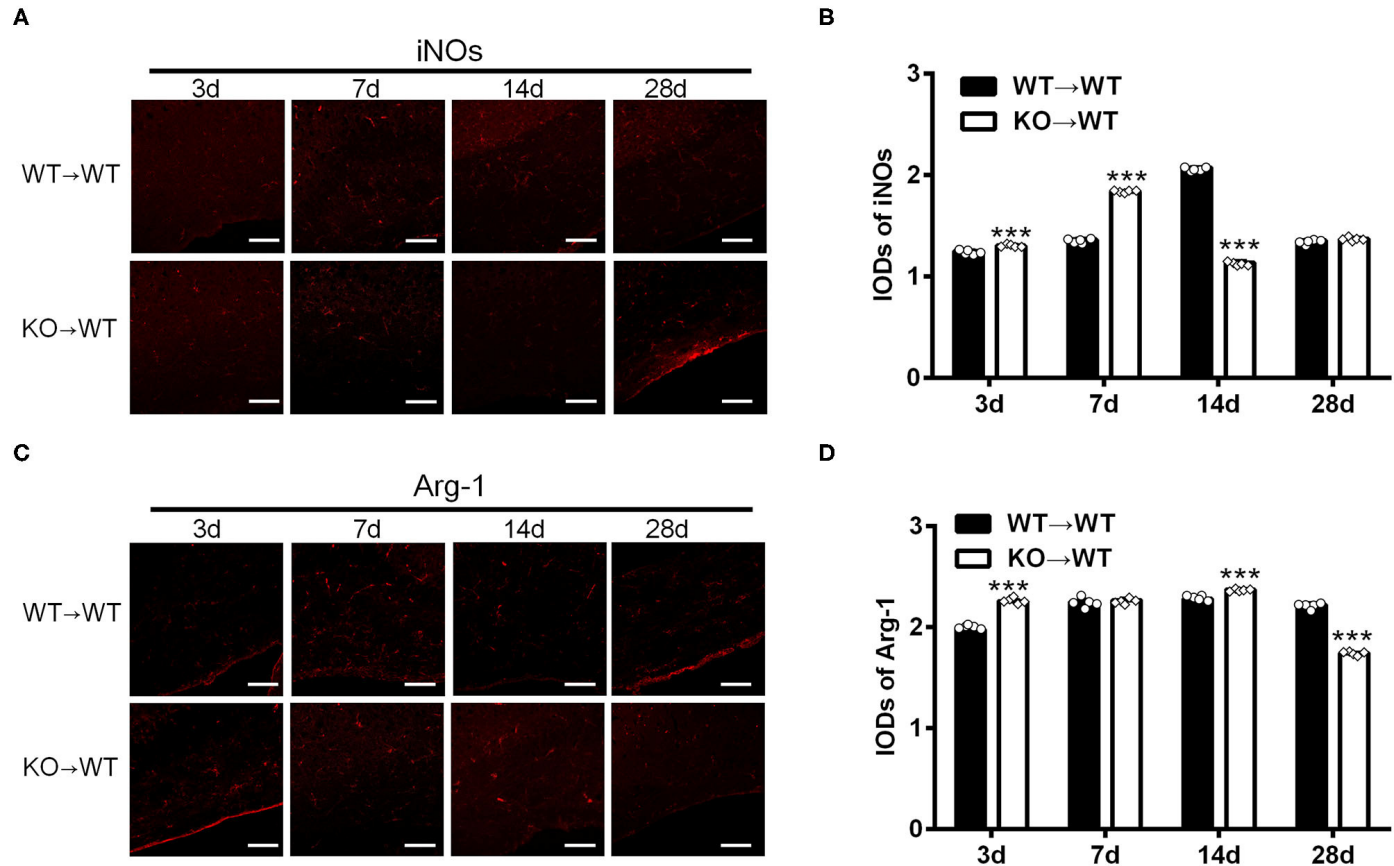
A_2A_R in BMDCs modulated the expression of iNOs and Arg-1 in corpus callosum in mice underwent chronic cerebral hypoperfusion. **(A,C)** Representative images showing the expression of iNOs and Arg-1 in control mice (WT → WT) and chimeric mice (KO → WT) established by bone marrow transplantation at 3, 7, 14, and 28 d after BCAS. Scale bars: 50 μm. **(B,D)** Statistical analysis showing the IOD for iNOs in chimeric mice was increased at 3 and 7 d but decreased after BCAS (***P < 0.001, Two-way ANOVA test followed by Turkey's multiple comparisons test. *n* = 5 mice for each group). The IOD for Arg-1 was stronger at 3 and 14 d in chimeric mice and decreased significantly at 28 d compared to the control mice (***P < 0.001, Two-way ANOVA test followed by Turkey's multiple comparisons test. *n* = 5 mice for each group).

To elucidate the effect of A_2A_R in BMDCs on microglia/macrophage polarization after chronic cerebral hypoperfusion, we performed BCAS in the chimeric mice where A_2A_R was selectively inactivated in BMDCs by bone marrow cell transplantation (Methods). Immunofluorescence staining for iNOs and Arg-1 in the corpus callosum of chimeric mice (KOWT) and control mice (WT → WT) was performed at 3, 7, 14, and 28 d after BCAS. As shown in the representative images and statistical analysis (Figures 3A,B), the IODs of iNOs in the corpus callosum were greater in the chimeric mice at 3 and 7 d after BCAS than the control mice, reduced at 14 d and back to the similar level within control mice at 28 d (^***^P < 0.001, Two-way ANOVA test followed by Turkey's multiple comparisons test). Similar analyses found that the IODs of Arg-1 in the corpus callosum were stronger in the chimeric mice at 3 and 14 d after BCAS and reduced significantly at 28 d compared to the control mice (^***^P < 0.001, Two-way ANOVA test followed by Turkey's multiple comparisons test; Figures 3C,D). Thus, as the IODs of both iNOs and Arg-1 were increased within 14 d after BCAS in the chimeric mice, and the IOD of Arg-1 was reduced while the IOD of iNOs was not affected at 28 d after BCAS, we reasoned that the selective inactivation of A_2A_R in BMDCs might polarize microglial/macrophage toward M1 phenotype in the corpus callosum at the later phase of white matter damage.

### A_2A_R Modulated the Polarization of Cultured Macrophages and Their Cytokine Expression

**Figure 4:**
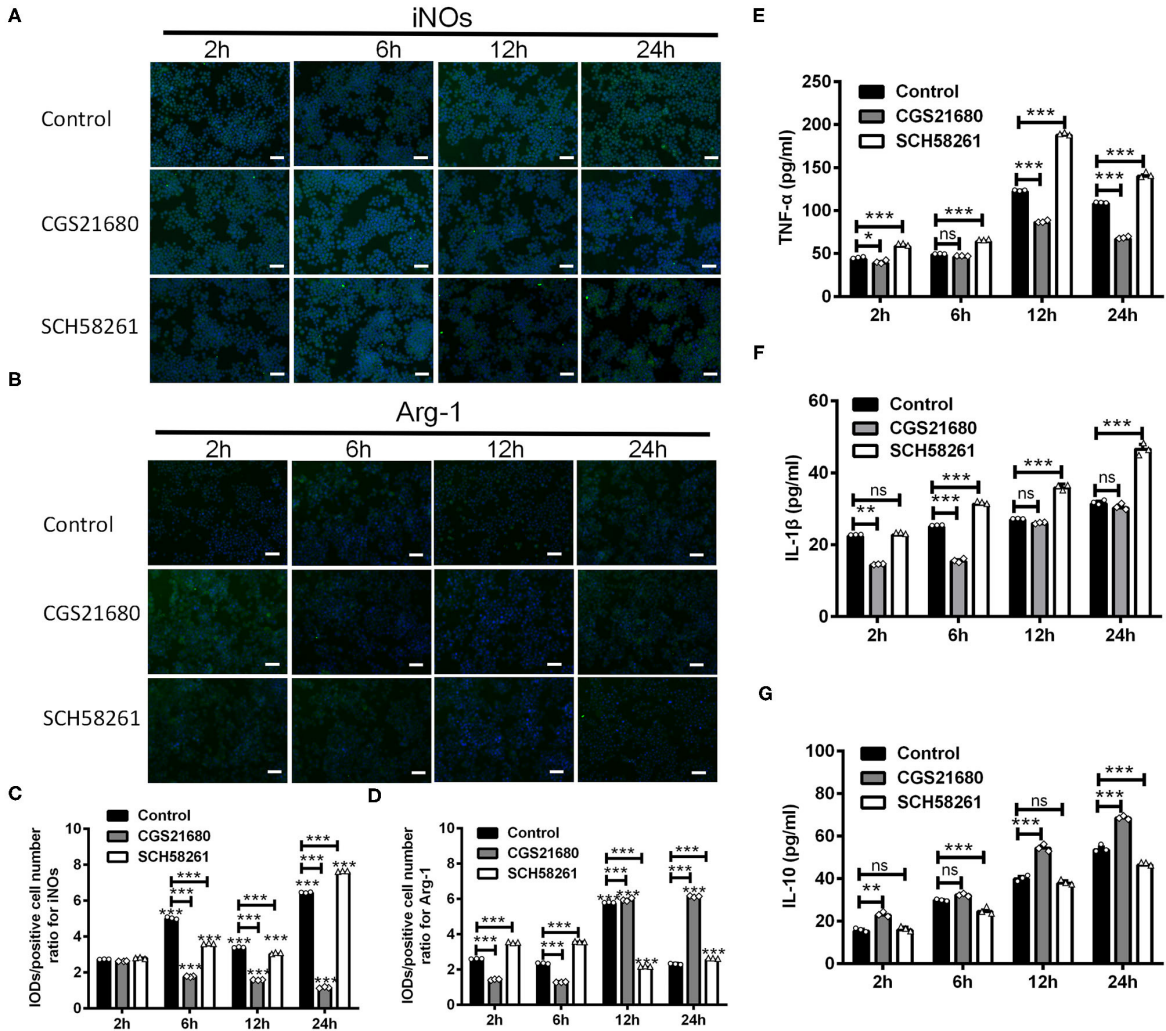
CGS21680 or SCH58261 regulated the expression of iNOs and Arg-1 in macrophages cultured in low-glucose and hypoxic conditions. **(A,B)** Representative immunofluorescent images for iNOs and Arg-1 in agonist GS21680 or SCH58261 treated macrophages at post-culture 2, 6, 12, and 24 h. Scale bars: 100 μm. **(C,D)** Statistically analysis showing the ratio of IOD/positive cells number for iNOs was significantly reduced by CGS21680 and increased by SCH5826 at post-culture 6, 12, and 24 h (***P < 0.001, Two-way ANOVA test followed by Turkey's multiple comparisons test. *n* = 3 independent experiments for each group). The IOD/positive cell number ratios for Arg-1 in the CGS21680 group were reduced at post-culture 2 and 6 h, but significantly increased at post-culture 12 and 24 h (***P < 0.001, Two-way ANOVA test followed by Turkey's multiple comparisons test. *n* = 3 independent experiments for each group). **(E,F)** Statistically analysis showing CGS21680 or SCH58261 inhibited or potentiated the increase of the protein of TNF-α, IL-1β at post-culture (ns: *P* > 0.05, *P < 0.05, **P < 0.01, ***P < 0.001, Two-way ANOVA test followed by Turkey's multiple comparisons test. *n* = 3 independent experiments for each group). **(G)** Statistically analysis showing the protein expression of IL-10 was increased after post-culture, while CGS21680 or SCH58261 exacerbated or inhibited the expressional level of IL-10 (ns: *P* > 0.05, **P < 0.01, ***P < 0.001, Two-way ANOVA test followed by Turkey's multiple comparisons test. *n* = 3 independent experiments for each group). ns, non significant; *p* >0.05.

To test whether A_2A_R activation could switch macrophages from M1 phenotype to M2 phenotype, we treated RAW264.7 macrophage cells with the A_2A_R agonist CGS21680 or antagonist SCH58261 and cultured the cells under low-glucose and hypoxic conditions. For both drug treatments, we used the dose of 1.0 μM, which was shown to be sufficient to modulate the protein and mRNA expression of iNOS, CD16/23, Arg-1 and CD206 in such culture conditions (Supplementary Figure 1). The expression of iNOs and Arg-1 was measured at 2, 6, 12, and 24 h after cultured under low-glucose and hypoxic conditions (referred to as “post-culture” hereafter). Representative results for iNOs immunostaining in macrophages were shown in Figure 4A. Statistical analysis showed that the IOD/positive cell number ratios for iNOs were reduced by CGS21680 while increased by SCH58261 at post-culture 6, 12, and 24 h (^***^P < 0.001, Two-way ANOVA test followed by Turkey's multiple comparisons test; Figure 4C). Representative results for Arg-1 immunostaining were shown in Figure 4B. Statistical analysis showed the IOD/positive cell number ratios for Arg-1 in the CGS21680 group were reduced than the control group at post-culture 2 and 6 h, but significantly increased at post-culture 12 and 24 h (^***^P < 0.001, Two-way ANOVA test followed by Turkey's multiple comparisons test; Figure 4D). With SCH58261 treatment, the ratios for Arg-1 were increased at post-culture 2 and 6 h, and reduced significantly at post-culture 12 and 24 h (^***^P < 0.001 vs. 2 h in the same group, Two-way ANOVA test followed by Turkey's multiple comparisons test; Figure 4D). Together, these findings suggest that the expression of iNOs is down-regulated and the expression of Arg-1 is up-regulated by CGS21680 ultimately in the chronic phase of low-glucose and hypoxic conditions.

In addition, we evaluated the protein expression of TNF-α, IL-1β, and IL-10 in macrophages upon the CGS21680 or SCH58261 treatment by ELISA. Statistical results showed that for the control group, the protein levels of inflammatory cytokines TNF-α and IL-1β were increased along with the culture time, and the treatment of CGS21680 or SCH58261 inhibited or potentiated the increasing tendency, respectively (ns: *P* > 0.05, ^*^P < 0.05, ^**^P < 0.01, ^***^P < 0.001, Two-way ANOVA test followed by Turkey's multiple comparisons test; Figures 4E,F). Conversely, for the anti-inflammatory cytokine IL-10, while the protein level was also increased along with the culture time, SCH58261 and CGS21680 displayed inductory and inhibitory effects, respectively (ns: *P* > 0.05, ^**^P < 0.01, ^***^P < 0.001, Two-way ANOVA test followed by Turkey's multiple comparisons test; Figure 4G). Taken together, these results suggest that inflammatory and anti-inflammatory cytokines in macrophages cultured under low-glucose and hypoxic conditions was likely to be modulated by the A_2A_R in the opposite directions.

### PPARγ-P65 Axis Was Involved in Macrophages Polarization in Low-Glucose and Hypoxic Conditions

**Figure 5:**
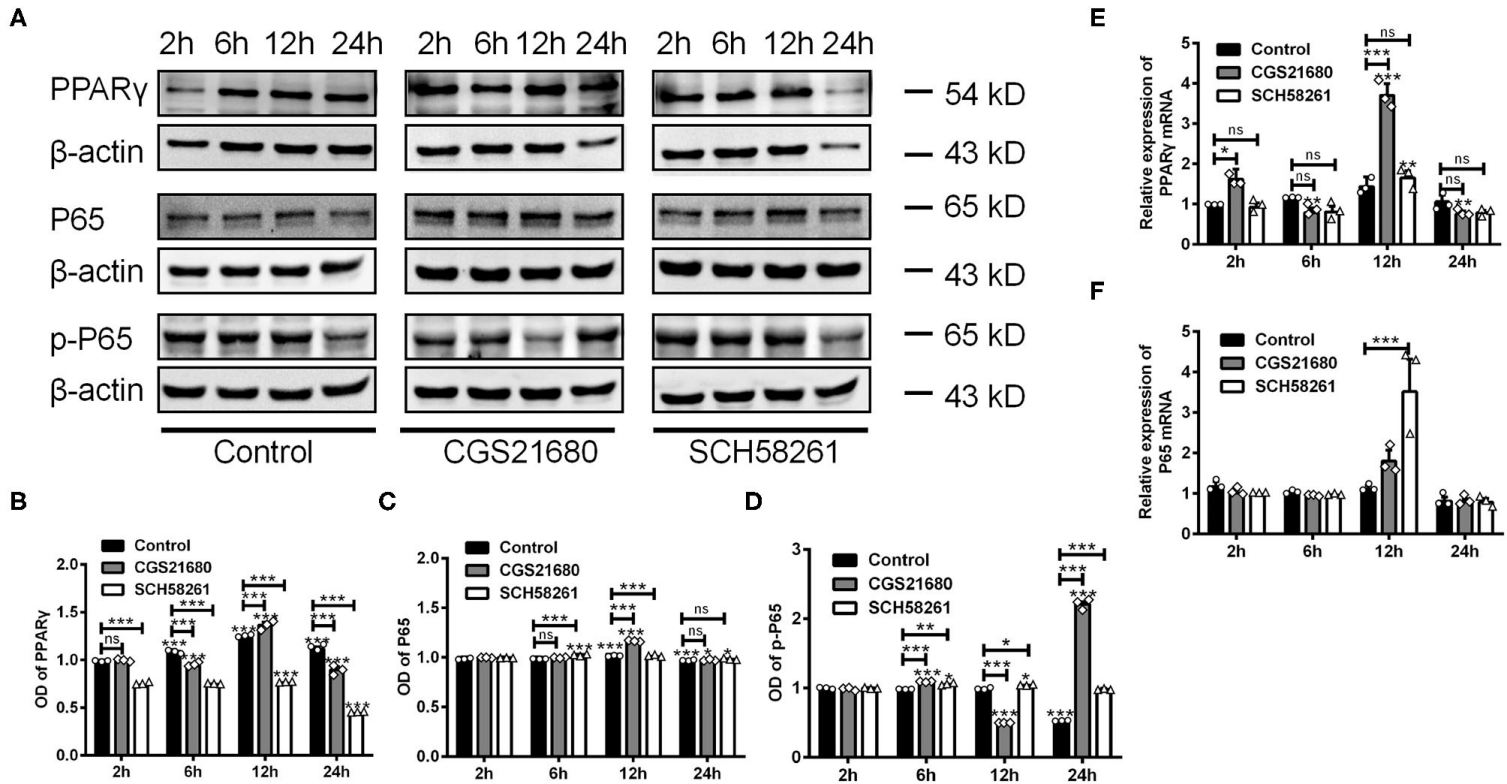
The expression of PPARγ, P65, and p-P65 was altered in macrophages cultured in low glucose and hypoxia. **(A)** Representative electrophoretic bands showing the expression of PPARγ, P65 and p-P65 in control, CGS21680 and SCH58261 treated macrophages at post-culture. β-actin was used as internal control. **(B)** Statistical analysis showing the expression of PPARγ in macrophages was increased gradually in low glucose and hypoxic conditions, and was increased further by CGS21680 at post-culture 12 h, but SCH58261 reduced the expression of PPARγ at post-culture (ns: *P* > 0.05, ***P < 0.001. Two-way ANOVA test followed by Turkey's multiple comparisons test. *n* = 3 independent experiments for each group). **(C)** Statistical analysis showing the expression of P65 was increased in macrophages at post-culture 12 and 24 h, and the expression was further increased by CGS21680 at 12 h and SCH58261 at 6 and 12 h (ns: *P* > 0.05, ***P < 0.001. Two-way ANOVA test followed by Turkey's multiple comparisons test. *n* = 3 independent experiments for each group). **(D)** Statistical analysis showing the expression of p-P65 was increased by CGS21680 at post-culture 6 and 24 h, and was increased in SCH58261 treated macrophages at post-culture 6, 12, and 24 h (*P < 0.05, ***P < 0.001. Two-way ANOVA test followed by Turkey's multiple comparisons test. *n* = 3 independent experiments for each group). **(E,F)** Statistical analysis showing the mRNA of *PPAR*γ was increased in CGS21680 treated macrophages at 2 and 12 h, but not affected by SCH58261. The expression of *P65* mRNA was increased in SCH58261 treated macrophages at post-culture 12 h (ns: *P* > 0.05, *P < 0.05, **P < 0.01, ***P < 0.001. Two-way ANOVA test followed by Turkey's multiple comparisons test. *n* = 3 independent experiments for each group). ns, non significant; *p* >0.05.

Next, we examined whether A_2A_R-mediated macrophages polarization involves the PPARγ-P65 signaling pathway, which is known to mediate inflammatory responses in various diseases (Villapol, 2018). By western blotting, we found that the protein expression of PPARγ was increased at 6, 12, and 24 h post low-glucose and hypoxic culture for the control group (^***^P < 0.001 vs. 2 h, Two-way ANOVA test followed by Turkey's multiple comparisons test; Figures 5A,B). Upon CGS21680 treatment, the protein level of PPARγ was increased only at post-culture 12 h. In comparison, the treatment of SCH58261 led to the reduced expression of PPARγ at all the time points (^***^P < 0.001, Two-way ANOVA test followed by Turkey's multiple comparisons test; Figures 5A,B). Similarly, the expression of P65 was increased by CGS21680 at post-culture 12 and 24 h, and further increased by SCH58261 at post-culture 6 and 12 h (ns: *P* > 0.05, ^*^P < 0.05, ^***^P < 0.001, Two-way ANOVA test followed by Turkey's multiple comparisons test) (Figure 5C). The expression of p-P65 was increased by CGS21680 significantly at post-culture 6 and 24 h, though a slight reduction was observed at post-culture 12 h (^***^P < 0.001, Two-way ANOVA test followed by Turkey's multiple comparisons test; Figure 5D). Conversely, the expression of p-P65 was increased in SCH58261 treated macrophages at post-culture 6, 12, and 24 h (^*^P < 0.05, ^**^P < 0.01, ^***^P < 0.001, Two-way ANOVA test followed by Turkey's multiple comparisons test; Figure 5D).

RT-PCR results showed that the mRNA expression of PPARγ was altered in consistent with protein in either CGS21680 or SCH58261 treated macrophages (ns: *P* > 0.05, ^*^P < 0.05, ^**^P < 0.01, ^***^P < 0.001, Two-way ANOVA test followed by Turkey's multiple comparisons test; Figure 5E). However, the mRNA expression of P65 was increased in SCH58261 treated macrophages at post-culture 12 h (^***^P < 0.001, Two-way ANOVA test followed by Turkey's multiple comparisons test; Figure 5F). Taken together, these results suggest that the PPARγ-P65 signaling pathway is involved in A_2A_R modulated polarizing process of macrophages in low-glucose and hypoxic conditions, while more detailed roles of P65 and p-P65 remain to be further clarified.

### A_2A_R Facilitated the Switching of Macrophages From M1 Phenotype to M2 Phenotype and Increased IL-10 Expression *via* the PPARγ-P65 Pathway

Next, we used lentivirus-mediated shRNA knockdown to further assess the role of PPARγ in the macrophage polarization under low-glucose and hypoxic environment. The expression of PPARγ was effectively reduced by the shRNA knockdown, as shown in Supplementary Figure 2. The macrophages with the shRNA transfection were subsequently measured with cell phenotype and cytokine production in a time series manner (i.e., 2, 6, 12, and 24 h after the exposure to low-glucose and hypoxic culture).

**Figure 6:**
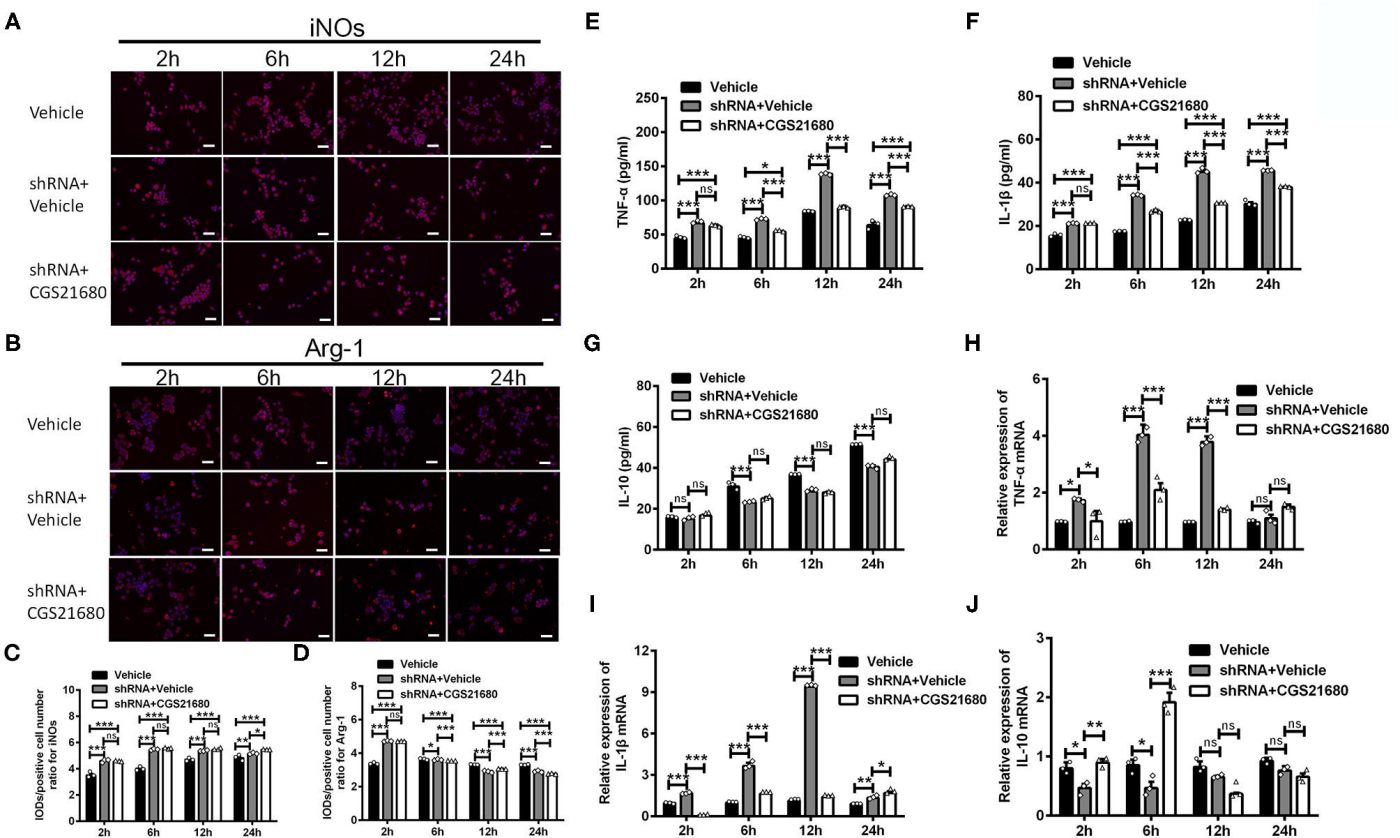
PPARγ knockdown affected the expression of cytokines in CGS21680 treated macrophages. **(A,B)** Representative images showing the expression of iNOs and Arg-1 in the macrophages pre-transfected with PPARγ shRNA and with or without CGS21680 treatment. Scale bars: 100 μm. **(C)** Statistical analysis showing the IOD/positive cells number for iNOs was increased in macrophages upon PPARγ knockdown at post-culture and was not reduced by CGS21680 (ns: *P* > 0.05, *P < 0.05, ***P < 0.001. Two-way ANOVA test followed by Turkey's multiple comparisons test. *n* = 3 independent experiments for each group). **(D)** Statistical analysis showing the IOD/positive cells number for Arg-1 was increased in shRNA+Vehicle group at post-culture 2 h, but was reduced at post-culture 6, 12, and 24 h, and was further reduced by CGS21680 at post-culture 6, 12, and 24 h (ns: *P* > 0.05, *P < 0.05, ***P < 0.001. Two-way ANOVA test followed by Turkey's multiple comparisons test. *n* = 3 independent experiments for each group). **(E,F)** ELISA results showing the expression of TNF-α and IL-1β was increased in macrophages upon PPARγ knockdown and was reduced by CGS21680 (ns: *P* > 0.05, *P < 0.05, ***P < 0.001. Two-way ANOVA test followed by Turkey's multiple comparisons test. *n* = 3 independent experiments for each group). **(G)** ELISA results showing the expression of IL-10 in macrophages was reduced after PPARγ knockdown and increased by CGS21680 (ns: *P* > 0.05, ***P < 0.001. Two-way ANOVA test followed by Turkey's multiple comparisons test. *n* = 3 independent experiments for each group). **(H,I)** RT-PCR results showing the mRNA expression of TNF-α and IL-1β was increased upon PPARγ knockdown and rescued by CGS21680 (ns: *P* > 0.05, *P < 0.05, ***P < 0.001. Two-way ANOVA test followed by Turkey's multiple comparisons test. *n* = 3 independent experiments for each group). **(J)** CGS21680 increased the mRNA expression of IL-10 in PPARγ shRNA pre-treated macrophages at post-culture 2 and 6 h (ns: *P* > 0.05, *P < 0.05, **P < 0.01, ***P < 0.001. Two-way ANOVA test followed by Turkey's multiple comparisons test. *n* = 3 independent experiments for each group). ns, non significant, *p* >0.05.

First, the polarizing state of macrophages was evaluated by immunofluorescence staining of iNOs and Arg-1. As shown by the representative images (Figure 6A) and statistical results (Figure 6C), the IOD/positive cell number ratios for iNOs were increased in macrophages with PPARγ knockdown at post-culture 2, 6, and 12 h (ns: *P* > 0.05, ^***^P < 0.0001, Two-way ANOVA test followed by Turkey's multiple comparisons test). Moreover, while CGS21680 alone significantly reduced the ratios of iNOs in macrophages under low-glucose and hypoxic condition, PPARγ knockdown abolished such negative impact of CGS21680 on iNOs (ns: *P* > 0.05, ^***^P < 0.001, Two-way ANOVA test followed by Turkey's multiple comparisons test). The IOD/positive cell number ratio for Arg-1 was increased at post-culture 2 h, but was decreased at post-culture 6, 12, and 24 h in shRNA+Vehicle group (^*^P < 0.05, ^***^P < 0.001, Two-way ANOVA test followed by Turkey's multiple comparisons test). Moreover, the ratio for Arg-1 was further reduced in shRNA+CGS21680 treated macrophages at post-culture 6, 12, and 24 h (ns: *P* > 0.05, ^***^P < 0.001, Two-way ANOVA test followed by Turkey's multiple comparisons test; Figures 6B,D), suggesting that activation of A_2A_R-induced upregulation of Arg-1 is modulated by PPARγ.

Second, the expression of inflammatory factors in macrophages was determined *via* ELISA and RT-PCR. Statistical results showed that the protein expression of both TNF-α and IL-1β were increased in macrophages with PPARγ knockdown after exposed in low-glucose and hypoxic conditions (^***^P < 0.001, Two-way ANOVA test followed by Turkey's multiple comparisons test; Figures 6E,F). Moreover, the expression of TNF-α and IL-1β in PPARγ shRNA and CGS21680 double treatment group was higher than that in vehicle group (^***^P < 0.001, Two-way ANOVA test followed by Turkey's multiple comparisons test; Figures 6E,F). Since CGS21680 treatment alone reduced the expression of TNF-α and IL-1β in macrophages, these results suggest that PPARγ knockdown antagonizes the negative impact of CGS21680 on the expression of these two cytokines. Meanwhile, the protein expression of IL-10 was reduced after PPARγ knockdown, and CGS21680 treatment did not rescue this reduction (ns: *P* > 0.05, ^***^P < 0.001, Two-way ANOVA test followed by Turkey's multiple comparisons test; Figure 6G). RT-PCR results further showed that the mRNA levels of TNF-α and IL-1β were altered in consistent to the protein (^*^P < 0.05, ^***^P < 0.001, Two-way ANOVA test followed by Turkey's multiple comparisons test; Figures 6H,I). The mRNA of IL-10 was reduced by PPARγ knockdown and increased by CGS21680 at post-culture 2 and 6 h, while the expression were not changed afterwards (ns: *P* > 0.05, ^*^P < 0.05, ^**^P < 0.01, ^***^P < 0.001, Two-way ANOVA test followed by Turkey's multiple comparisons test; Figure 6J).

**Figure 7:**
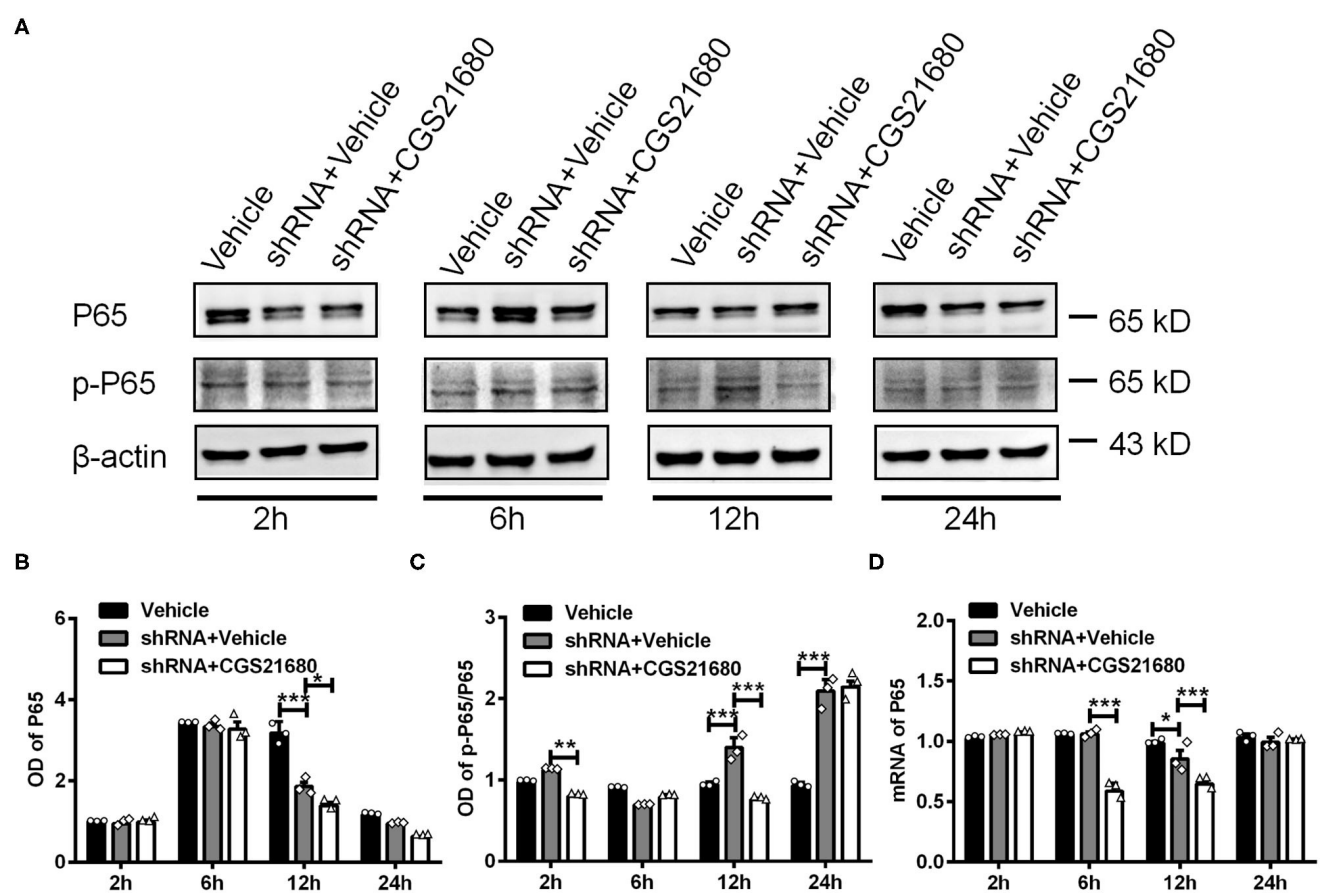
PPARγ regulated the expression of P65 and p-P65 in CGS21680 treated macrophages. **(A)** Representative electrophoretic bands showing the expression of P65 and p-P65 in PPARγ shRNA pre-transfected macrophages with or without CGS21680 treatment after cultured in low glucose and hypoxic conditions. **(B,C)** Densitometric analysis results showing the expression of P65 were reduced in shRNA targeted macrophages and further reduced by CGS21680 at post-culture 12 h (*P < 0.05, ***P < 0.001. Two-way ANOVA test followed by Turkey's multiple comparisons test. *n* = 3 independent experiments for each group). **(C)** The ratio of p-P65/P65 was increased in macrophages upon PPARγ knockdown at post-culture 12 and 24 h, but was reduced in by additional CGS21680 treatment at post-culture 2 and 12 h (***P < 0.001. Two-way ANOVA test followed by Turkey's multiple comparisons test. *n* = 3 independent experiments for each group). **(D)** RT-PCR results showing the mRNA expression of P65 was reduced in macrophages upon PPARγ knockdown at post-culture 12 h and was potentiated by additional CGS21680 treatment at post-culture 6 and 12 h (*P < 0.05, ***P < 0.001. Two-way ANOVA test followed by Turkey's multiple comparisons test. *n* = 3 independent experiments for each group). **P < 0.01.

Finally, we examined the expression of P65 and p-P65 in macrophages upon PPARγ knockdown and/or CGS21680 treatment. Western blotting showed that the protein expression of P65 was decreased upon PPARγ knockdown at post-culture 12 h, and further reduced by additional CGS21680 treatment at post-culture 12 and 24 h (^*^P < 0.05, ^***^P < 0.001, Two-way ANOVA test followed by Turkey's multiple comparisons test; Figures 7A,B). Moreover, the ratio of p-P65 to P65 was increased in shRNA+Vehicle group at post-culture 12 and 24 h, but CGS212680 reduced the ratio of p-P65/P65 in PPARγ knockdown macrophages at post-culture 2 and 12 h (^**^P < 0.01, ^***^P < 0.001, Two-way ANOVA test followed by Turkey's multiple comparisons test; Figure 7C). Consistently, the mRNA level of P65 was significantly decreased in the shRNA+Vehicle group at post-culture 12 h, and further reduced in shRNA+CGS21680 group at post-culture 6 and 12 h (^*^P < 0.05, ^***^P < 0.001, Two-way ANOVA test followed by Turkey's multiple comparisons test; Figure 7D). Taken together, these results suggest that PPARγ-P65 pathway is involved in A_2A_R-induced macrophage M2 polarization and inflammatory responses.

## Discussion

Here, our study combined mouse models and macrophage cell lines to provide evidence that A_2A_R in BMDCs is likely to modulate macrophages polarization in white matter lesions induced by chronic cerebral hypoperfusion. Moreover, our PPARγ knockdown experiments further suggest that PPARγ-P65 pathway might be significantly involved in A_2A_R-associated neuroprotective effect in white matter lesions.

Aberrant phenotypical activation of microglia/macrophages has been shown to disrupt normal tissue morphology, phagocytosis capacity, secretion of cytokines and lead to various CNS disorders, such as ischemic stroke (Qin et al., 2017, 2018, 2019; Jiang et al., 2020; Yang et al., 2021), intracerebral hemorrhage (Lan et al., 2017), multiple sclerosis (Zia et al., 2020), traumatic brain injury (Yang et al., 2019), traumatic spinal cord injury (Liu et al., 2020), demyelination diseases (Aryanpour et al., 2017; Chu et al., 2018), and Alzheimer's disease (Jin et al., 2020; Ren et al., 2020). Recently, it has been shown that the M1/M2-based phenotypical definition for this cell group might be vague. For instance, single-cell RNA-seq analysis has showed that the canonical “M1-like” and “M2-like” gene expression profiles were highly overlapped in the macrophages isolated from traumatic brain tissues (Kim et al., 2016). Therefore, one limitation of our study could be the concept of M1 and M2 phenotypes we sought to define based on several selected molecular markers. Nonetheless, we think that under the context of CNS inflammatory response, the switched expression between iNOs and CD16/32 (“M1-like” phenotype markers), and Arg-1 and CD206 (“M2-like” phenotype markers), together with the corresponding alteration of pro- and anti-inflammatory cytokine production, point to a specific microglial/macrophage polarization. To what degree does such a polarization reflects the molecular boundaries of the classical M1/M2 model, or it actually indicates a novel phenotypic dynamics would be an interesting research topic to pursue in our following studies.

A_2A_R signaling affects the pathology of a range of neurological disorders (Ahmad et al., 2017b, 2018a; Ansari et al., 2017b; Carvalho et al., 2019; Chen et al., 2020). Our previous studies showed that A_2A_R deletion aggravates white matter damage and A_2A_R in BMDCs is an important modulator of white matter lesions induced by chronic cerebral hypoperfusion (Duan et al., 2009; Ran et al., 2015). To further investigate the neuroprotective effect of A_2A_R activation in this pathological process, we generated chimeric mice with selective inactivation of A_2A_R in BMDCs through bone marrow cell transplantation and assessed the state of microglia, inflammatory cytokine expression. We found that activation of A_2A_R in BMDCs induced the polarization of microglia/macrophages towarding M2 phenotype in white matter lesions. In addition, we showed that A_2A_R agonist and antagonist effectively regulated inflammatory responses in macrophages after exposure to low-glucose and hypoxic conditions. These results suggest that A_2A_R in BMDCs is involved in switching macrophages from M1 phenotype to M2 phenotype under low-glucose and hypoxic conditions.

The mechanisms underlying macrophage M2 polarization have not been fully elucidated. Several studies have reported the expression of PPARγ was increased when macrophages was polarized to M2 phenotype by stimulation with IL-4 (Zhou et al., 2020), procyanidin B2 (Tian et al., 2019), or malibatol A (Pan et al., 2015). Consistent with previous studies (He et al., 2017; Huang et al., 2019), here we showed that CGS21680 induced macrophage M2 polarization could be reversed by knockdown of PPARγ with shRNA. In addition, we found that the expression of P65, the downstream molecule of PPARγ, was reduced in macrophages upon PPARγ knockdown. Together, these results suggest that the PPARγ-P65 pathway is involved in the A_2A_R-mediated M1 to M2 macrophage phenotypical switching in low-glucose and hypoxic conditions.

PPARγ exerts neuroprotective effect by regulating the expression of pro-inflammatory or anti-inflammatory factors in brain injuries or brain ischemia (Maréchal et al., 2018; Villapol, 2018). We showed that the expression of TNF-α and IL-1β was increased, whereas the expression of IL-10 was reduced in macrophages transfected with PPARγ shRNA. Further, knockdown of PPARγ efficiently antagonized the effect of CGS21680 on reducing inflammatory cytokines and increasing anti-inflammatory factors, suggesting that the PPARγ-P65 pathway might also participate in the modulatory effect of A_2A_R on macrophages polarization and cytokine production under low-glucose and hypoxic conditions.

## Conclusion

In summary, our results characterized the potential role of A_2A_R in bone marrow-derived cells in modulating macrophage polarization *via* PPARγ-P65 signaling axis in the white matter damage induced by chronic hypoperfusion.

## Data Availability Statement

The original contributions presented in the study are included in the article/Supplementary Material, further inquiries can be directed to the corresponding author/s.

## Ethics Statement

The animal study was reviewed and approved by the Institutional Animal Care and Use Committee of the Army Medical University. Written informed consent was obtained from the owners for the participation of their animals in this study.

## Author Contributions

K-JM, K-FS, and WD contributed to conception, study design, draft, and revise the manuscript and figures. K-JM, K-FS, Y-LL, Z-FW, and WD contributed to acquisition and analysis of data, verify the underlying data, interpretation of results, and preparation of figures. All authors edited and approved the paper.

## Funding

This work was supported by grants from the National Natural Science Foundation of China (No. 81873757) and the Research Foundation of Army Military Medical University (2019XLC3030).

## Conflict of Interest

The authors declare that the research was conducted in the absence of any commercial or financial relationships that could be construed as a potential conflict of interest.

## Publisher's Note

All claims expressed in this article are solely those of the authors and do not necessarily represent those of their affiliated organizations, or those of the publisher, the editors and the reviewers. Any product that may be evaluated in this article, or claim that may be made by its manufacturer, is not guaranteed or endorsed by the publisher.
