# Supplementary material for: Adenosine A2A Receptor in Bone Marrow-Derived Cells Mediated Macrophages M2 Polarization via PPARγ-P65 Pathway in Chronic Hypoperfusion Situation
Source: Front Aging Neurosci. 2022 Jan 3;13:792733. doi: 10.3389/fnagi.2021.792733 (PMC8761901; doi:10.3389/fnagi.2021.792733)
Supplement: Supplementary file 1 [file Data_Sheet_1.docx]

**Supplemental Materials**

# Supplementary Tables

Table 1 Primer Characteristics

|  | Sense Primer (5’-3’) | Antisense Primer (5’-3’) |
| --- | --- | --- |
| PPARγ | GGCCGAGAAGGAGAAGCTGTTG | GGCCACCTCTTTGCTCTGCTC |
| P65 | CGGGATGGCTACTATGAGGCTG | CCCGCACTGTCACCTGGAAGC |
| TNF-α | CGCTGAGGTCAATCTGCCCAAGTAC | GGGGGCTGGGTAGAGAATGGATG |
| IL-1β | CTCGTGCTGTCGGACCCATATG | GTGGGTGTGCCGTCTTTCATTAC |
| IL-10 | AAGGCAGTGGAGCAGGTGAAGAG | GCAGGTGTTTTAGCTTTTCATTTTG |
| β-actin | ACCCCGTGCTGCTGACCGAG | TCCCGGCCAGCCAGGTCCA |

# Supplementary Figures

1. The appropriate concentrations of CGS21680 and SCH58261 were determined by western blotting in the preliminary experiment. As shown in Supplementary Figure 1, the protein expression of M1 or M2 markers was increased in macrophages after post-cultured in low-glucose and hypoxic conditions, and was altered by CGS21680 or SCH58261 most significantly at 1.0 μM (****P* < 0.001, Two-way ANOVA test followed by Turkey’s multiple comparisons test).

**
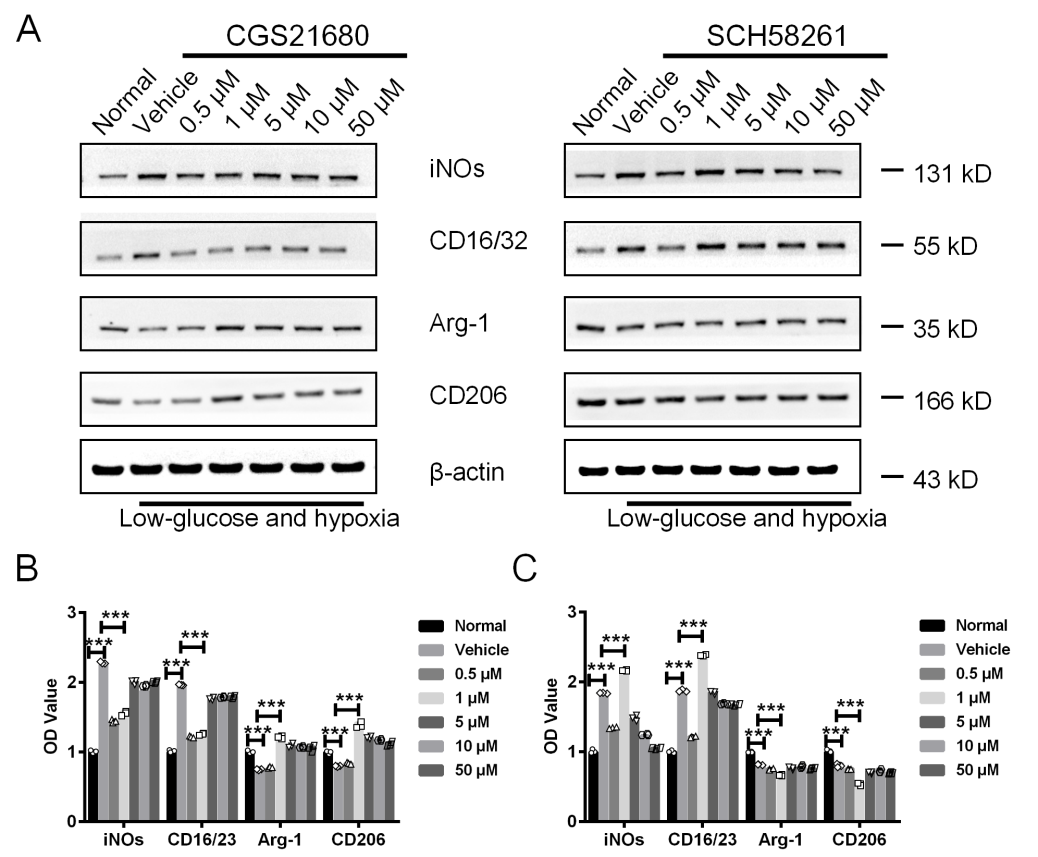
**

Supplementary Figure 1. The protein expression of iNOs, CD16/32, Arg-1 and CD206 in macrophages treated with CGS21680 or SCH58261 at different concentrations in low glucose and hypoxia conditions. A. Representative protein electrophoretic bands of iNOs, CD16/32, Arg-1 and CD206 in CGS21680 or SCH58261 treated macrophages. β-actin was used as internal control. B. Statistical results showing the protein expression of iNOs and CD16/32 was increased in macrophages after post-culture, while was significantly reduced by 1.0 μM CGS21680. Meanwhile, the protein expression of Arg-1 and CD206 was reduced in macrophages after post-culture, but was significantly increased by 1.0 μM CGS21680. C. Statistical results showing the protein expression of iNOs and CD16/32 was increased in macrophages after post-culture, and the expression of Arg-1 and CD206 was reduced in macrophages after post-culture, and these effects were potentiated by 1.0 μM SCH58261. ****P* < 0.001, Two-way ANOVA test followed by Turkey’s multiple comparisons test. n = 3 independent experiments for each group.

2. The efficiency of *PPARγ* shRNA was assessed by western blotting and PCR. As shown in Supplementary Figure 2, the protein and mRNA level of PPARγ in macrophages was reduced most significantly by PPARγ-Mus-1024 (ns: *P* > 0.05, ****P* < 0.001, Unpaired two-tailed t test).


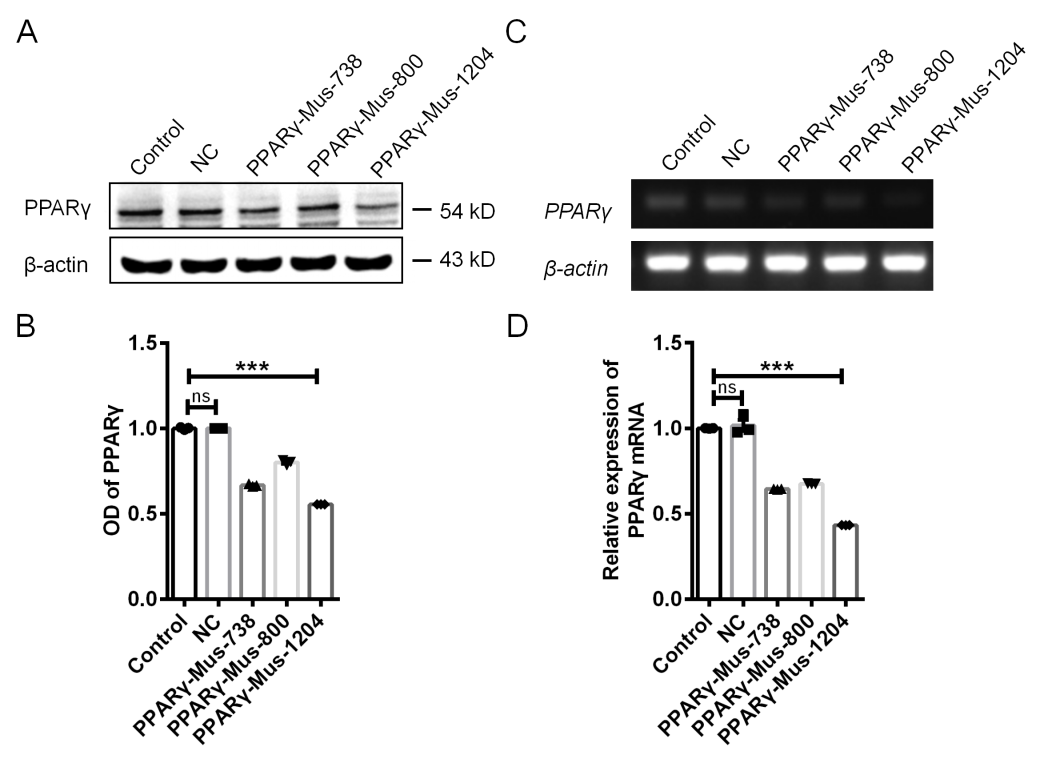


Supplementary Figure 2. The protein and mRNA level of PPARγ in transfected macrophages transfected with lentivirus carrying PPARγ shRNA. A. Representative electrophoretic bands showing the protein of PPARγ and β-actin in Control, negative control (NC, 5’-3’: TTCTCCGAACGTGTCACGT), PPARγ-Mus-738 (5’-3’: GGATGTCTCACAATGCCATCA), PPARγ-Mus-800 (5’-3’: GCGGAGATCTCCAGTGATATC) and PPARγ-Mus-1204 (5’-3’: GCTGGCCTCCCTGATGAATAA) treated macrophages. B. Statistical results showing the optical density of PPARγ was reduced by PPARγ-Mus-1204 most significantly. ns: *P* > 0.05, ****P* < 0.001, Unpaired two-tailed t test. n = 3 independent experiments for each group. C. Representative bands showing the mRNA of *PPARγ* and *β-actin* in Control, negative control (NC), PPARγ-Mus-738, PPARγ-Mus-800 and PPARγ-Mus-1204 treated macrophages. D. Statistical results showing the expression of *PPARγ* was reduced by PPARγ-Mus-1204 most significantly. ns: *P* > 0.05, ****P* < 0.001, Unpaired two-tailed t test. n = 3 independent experiments for each group.
